# Complete genome sequence of *Clostridioides difficile* NCTC 12727, a PCR ribotype 001 reference strain

**DOI:** 10.1128/mra.00589-26

**Published:** 2026-07-20

**Authors:** Samiksha Venkatesan, Michael Macey, Uzaira Saniah, Ernesto Abel-Santos, Rachel McMullan, Ilias Kounatidis, Terry Bilverstone

**Affiliations:** 1 Anaerobes in Medicine (AiM Lab), School of Life, Health and Chemical Sciences, The Open University 5488, Milton Keynes, United Kingdom; 2 AstrobiologyOU, School of Environment, Earth and Ecosystem Sciences, The Open University 5488, Milton Keynes, United Kingdom; 3 Department of Chemistry and Biochemistry, University of Nevada–Las Vegas 14722, Las Vegas, Nevada, USA; 4 Nevada Institute of Personalized Medicine, University of Nevada–Las Vegas 14722, Las Vegas, Nevada, USA; 5 School of Life, Health and Chemical Sciences, The Open University 5488, Milton Keynes, United Kingdom; Queens College Department of Biology, Queens, New York, USA

**Keywords:** *Clostridium difficile*, genomics, mobile genetic elements

## Abstract

We report the complete genome sequence of the PCR ribotype 001 *Clostridioides difficile* strain NCTC 12727 obtained by hybrid genome assembly, manual curation, and PCR validation. The circular 4,371,963 bp genome (28.99% GC) contained two multi-copy mobile genetic elements encompassing a prophage and a Tn*916*-like element, respectively.

## ANNOUNCEMENT

*Clostridioides difficile* (formerly *Clostridium difficile* [1]) is the leading cause of hospital-associated diarrhea in the developed world. Only two antimicrobials are currently recommended for the treatment of *C. difficile* infection (CDI), and *C. difficile* is extensively drug resistant (2). Treatment failure and recurrent infection are common, highlighting the need for improved therapeutics.

PCR ribotype 001 (RT 001) strains are highly prevalent in Eastern European nations (3, 4), the Middle East, and Asia (5). *C. difficile* NCTC 12727 is a human fecal clinical isolate from St. Bartholomew’s Hospital (London, UK, isolated 1992). This reference RT 001 strain has been investigated for the therapeutic activity of its harbored bacteriophage φCD27 and the derived endolysin (6–8). Despite the phage genome being published, the genome sequence of the host strain has not been previously determined.

We acquired *C. difficile* NCTC 12727 from the National Collection of Type Cultures and propagated it on Brain Heart Infusion-supplemented (BHIS) medium (Oxoid) at 37°C under anaerobic conditions (85% N_2_, 10% CO_2_, and 5% H_2_) using an A95 Anaerobic Workstation (Don Whitley Scientific). An overnight culture was harvested, resuspended in DNA/RNA Shield (Zymo Research), and sent to MicrobesNG for DNA extraction and purification (solid-phase reversible immobilization beads) and multi-platform sequencing.

Illumina and Oxford Nanopore Technology (ONT) libraries were generated using Nextera XT and SQK-RBK114.96 kits, respectively, with individual barcoded ONT samples pooled into a single library using 200–400 ng of HMW DNA. The Illumina library was sequenced on a NovaSeq 6000 (250 bp paired-end), providing 587,951,507 bases after Trimmomatic v0.3 adapter trimming using a sliding window (Q15) (9). ONT sequencing on a GridION (R10.4.1 flow cell) generated 60,766 reads and 621,975,123 bases (*N*_50_: 16,777 bp, mean *Q*-score: 38.1). Base calling used Dorado model r1041_e82_400bps_sup_variant_v4.3.0 in super accuracy mode.

Hybrid assembly was performed using Unicycler v0.4.8 (10) under bold settings using unfiltered ONT reads, yielding six contigs with an *N*_50_ value of 1,339,619 bp (C1: 1,514,704 bp; C2: 1,339,619 bp; C3: 1,167,692 bp; C4: 179,104 bp; C5: 50,936 bp, and C6: 34,486 bp). Default parameters were used, except where otherwise noted.

**Fig 1:**
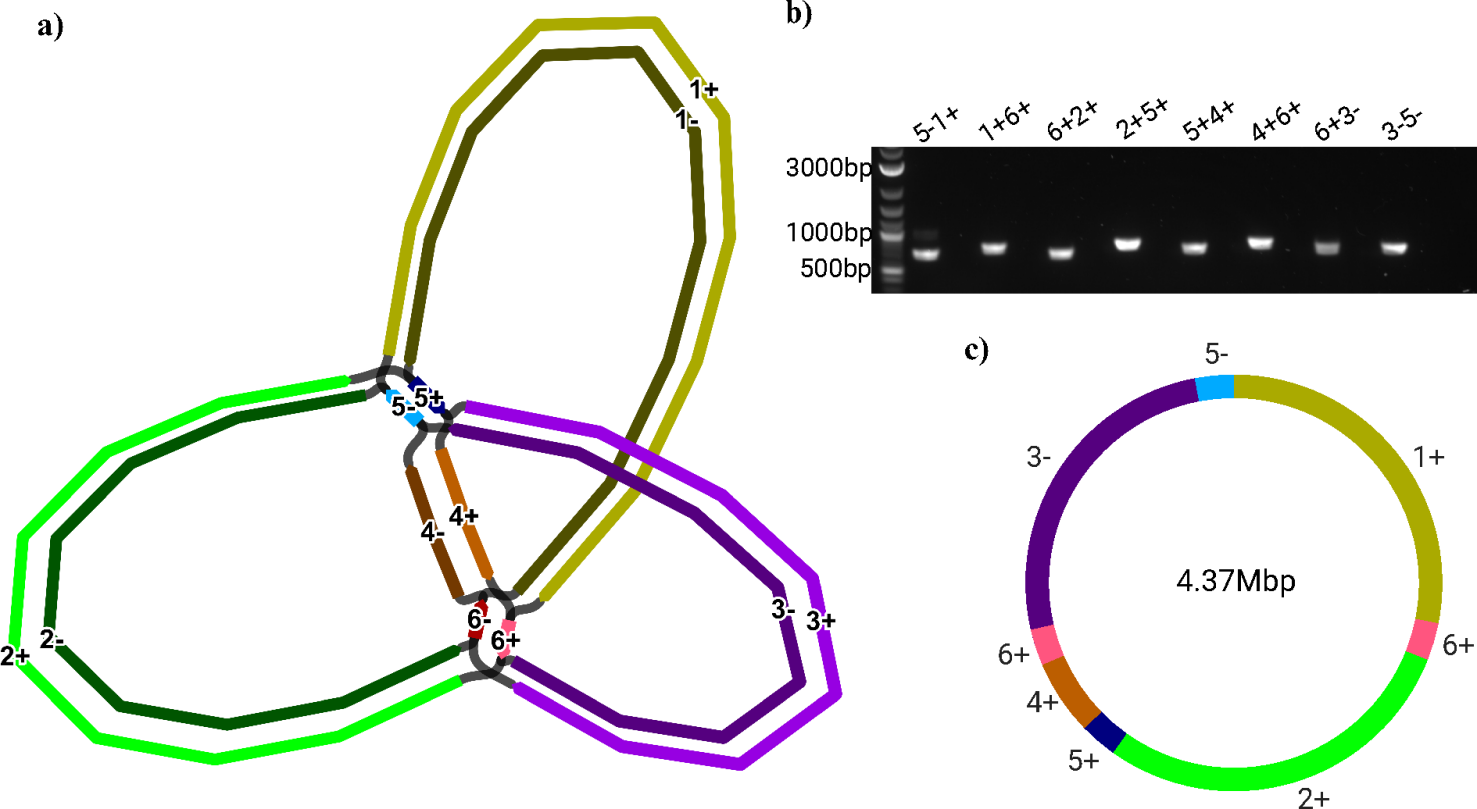
(**a**) Initial assembly graph visualized using Bandage; (**b**) gel displaying the junction-specific PCR amplicons electrophoresed on a 1% agarose gel. Each amplicon is followed by a negative control for the primer set in the adjacent lane. Primers (IDT DNA): C5-F GTCCCTATAGCTTTGCGTC, C1+R CTGTATAAAATGCAGTTATAGAACC, C1+F GTTTCGGGTATACTTATGTC, C6+R1 GTTTACTTTCTGCATGAACG, C6+F GTGCGAGGATTGATTAAG, C2+R CTTTCTTTTGATGGAATCAAGTC, C2+F GCTTCTTCTTGGTCATGTG, C5+R GAACAATATCTTTTTGGCAACTG, C5+F CATAAACATATGGTGAAATTAGAAG, C4+R CAATAAGTGATTCAGCTATGG, C4+F CTCTATATGTCTGGTAAACCTTTTC, C6+R2 CGAACCGTCATTCATTAC, C3-R CATGTTATTCATATCCATCCC, C3-F GGACTTACTGTAGAAGTTG, and C5-R CCACAATTACATAAACATATGGTG. (**c**) Representation of the complete genome map of *C. difficile* NCTC 12727 demonstrating the location and orientation (+/−) of the contigs, which are color-coded to match the Bandage assembly graph.

The genome structure was resolved by analyzing the assembly graph topology using Bandage v0.9.0 (11), which revealed tangles at contigs 5 (φCD27) and 6 (Tn*916*-like element) (Fig. 1a). These regions exhibited 2.07-fold and 1.64-fold higher than average chromosomal coverage, respectively, demonstrating that these mobile genetic elements were each present at two distinct loci. The assembly graph report showed that all six contigs had blunt ends with 0 overlap. A complete circular genome was manually assembled after reconciling the logical path from the assembly graph with a *de novo* long-read assembly using Flye v2.9.4 (12), in which the predicted contig–contig junctions were confirmed. Finally, these junctions were experimentally validated by junction-specific PCR (see detailed protocol) (Fig. 1b).

The complete genome sequence of 4,371,963 bp (28.99% guanine–cytosine [GC] content) is depicted in Fig. 1c. The coverage was determined as 135-fold for Illumina and 142-fold for ONT. Analysis using CheckM v1.0.18 (13) confirmed the genome to be 100% complete with 0% contamination, validating that the multiple insertions of the two contigs had not duplicated any essential genes. The assembly was rotated to the *dnaA* gene using DFAST v1.13 and annotated using the National Center for Biotechnology Information (NCBI) Prokaryotic Genome Announcement Pipeline v16.11, revealing 3,977 CDSs, 35 rRNAs, and 90 tRNAs.

## Data Availability

Project data were deposited to NCBI under BioProject number PRJNA1464271 and BioSample number SAMN59701171. Illumina (SRX33302917) and ONT reads (SRX33302918) were uploaded to the SRA. The Whole-Genome Shotgun project has been deposited at DDBJ/ENA/GenBank under accession number JBYDFQ000000000. The version described in this paper is version JBYDFQ010000001.1. The detailed junction PCR protocol was uploaded to protocols.io.
